# Diagnostic Accuracy of Immature Granulocyte Count for Early Sepsis Detection: A Comparative Evaluation With C-Reactive Protein and Procalcitonin

**DOI:** 10.7759/cureus.112064

**Published:** 2026-07-04

**Authors:** Rekha Taver, Viswadeep Gupta, Prachi Saxena, Juhi Sharma, Adarshika Yadav, Abhijeet Humne

**Affiliations:** 1 Department of Pathology, Veerangana Avanti Bai Lodhi Autonomous State Medical College, Etah, IND; 2 Department of General Surgery, Veerangana Avanti Bai Lodhi Autonomous State Medical College, Etah, IND; 3 Department of Pathology, Autonomous State Medical College, Pilibhit, IND; 4 Department of Pathology, Geetanjali Institute of Medical Sciences, Jaipur, IND; 5 Department of Orthodontics, Career Post Graduate Institute of Dental Science and Hospital, Lucknow, IND; 6 Department of Oral and Maxillofacial Surgery, Babu Banarasi Das College of Dental Sciences, Lucknow, IND

**Keywords:** c-reactive protein, immature granulocytes, procalcitonin, retrospective, sepsis

## Abstract

Introduction: Sepsis is a life-threatening condition characterized by a dysregulated host response to infection, resulting in organ dysfunction and high mortality rates among critically ill patients. Early recognition is essential for prompt therapeutic intervention; however, nonspecific clinical presentation often delays diagnosis. Conventional biomarkers, such as C-reactive protein (CRP) and procalcitonin (PCT), are frequently used, but their diagnostic performance varies. Immature granulocyte (IG) parameters obtained from routine hematological analyses have recently gained attention as potential early indicators of sepsis. This study aimed to evaluate the diagnostic accuracy of immature granulocyte count (IG#) and immature granulocyte percentage (IG%) for early sepsis detection and compare their performance with that of CRP and PCT in critically ill patients.

Materials and methods: This retrospective observational study included 178 critically ill patients admitted to the intensive care unit of a single institution. Laboratory investigations performed within 24 hours of admission included complete blood count with IG parameters, CRP, PCT, lactate, and other biochemical markers. Patients were monitored for the development of sepsis and subsequently categorized into sepsis and non-sepsis groups. The diagnostic performance was assessed using receiver operating characteristic (ROC) curve analysis.

Results: Of the 178 patients, 72 developed sepsis during the observation period. Patients with sepsis demonstrated significantly elevated IG#, IG%, PCT, CRP, lactate, white blood cell count, neutrophil percentage, serum creatinine, and bilirubin levels compared with patients without sepsis. ROC curve analysis revealed good diagnostic performance for IG%, followed by IG# and PCT, whereas CRP demonstrated a comparatively lower discriminatory ability. The combination of IG% and PCT achieved the best overall diagnostic performance, indicating improved accuracy in identifying patients at risk of developing sepsis. Elevated immature granulocyte parameters were strongly associated with sepsis and reflected early activation of the bone marrow response to infection.

Conclusion: Immature granulocyte parameters, particularly IG%, are reliable and readily available biomarkers for early sepsis detection. Their diagnostic performance is comparable to that of established markers and may be enhanced when combined with procalcitonin. Routine assessment of immature granulocytes may facilitate the early recognition of sepsis and support timely clinical decision-making in critically ill patients.

## Introduction

Sepsis is a life-threatening syndrome characterized by a dysregulated host response to infection, leading to organ dysfunction and substantial morbidity and mortality worldwide. Despite advances in critical care medicine, sepsis remains a major cause of intensive care unit (ICU) admissions and is associated with prolonged hospitalization, increased healthcare costs, and poor clinical outcomes [1]. Early identification and prompt initiation of appropriate therapy are crucial for improving survival, as delays in diagnosis can significantly increase the risk of organ failure and death [2]. However, the clinical presentation of sepsis is often nonspecific, making early diagnosis challenging, particularly for critically ill patients with multiple comorbidities.

Various laboratory biomarkers have been investigated to facilitate early detection of sepsis. Among these, C-reactive protein (CRP) and procalcitonin (PCT) are widely used in clinical practice because of their association with systemic inflammatory responses and bacterial infections [3]. Although these biomarkers provide valuable diagnostic information, their sensitivity and specificity may be influenced by several noninfectious conditions, limiting their utility as standalone indicators [4]. Consequently, there is an ongoing search for reliable, rapid, and cost-effective biomarkers that can improve diagnostic accuracy in critically ill patients.

Immature granulocytes, which include promyelocytes, myelocytes, and metamyelocytes, are released prematurely from the bone marrow during severe inflammatory and infectious processes and have emerged as promising markers of early infection [5]. Modern automated hematology analyzers can quantify immature granulocyte count (IG#) and percentage (IG%) as part of the routine complete blood count, providing rapid and inexpensive results without additional laboratory costs. Recent evidence suggests that elevated IG levels may precede conventional inflammatory markers and could serve as an early indicator of sepsis [6-8].

The aim of this study was to evaluate the diagnostic accuracy of IG# and IG% for the early detection of sepsis and to compare their performance with that of PCT and CRP in critically ill ICU patients. The objectives were to compare the diagnostic performance of immature granulocyte parameters with established biomarkers to determine the optimal diagnostic cut-off values, and to assess whether combining immature granulocyte parameters with PCT improves the prediction and identification of sepsis.

## Materials and methods

Study design and setting

This retrospective observational study was conducted in the Medical ICU of the Veerangana Avanti Bai Lodhi Autonomous State Medical College, Etah, India after obtaining approval from the Institutional Ethics Committee (VALASMC/IEC/2025/2675). Medical records of critically ill patients admitted to the ICU between July 2022 and January 2025 were reviewed. Written informed consent was obtained from the patients or their legally authorized representatives at the time of admission for the use of anonymized clinical information for research purposes. 

Study participants

A total of 178 eligible patients aged ≥ 15 years were included in the study. The patients were admitted or referred to the ICU for conditions other than sepsis. Individuals with evidence of sepsis at admission or who developed signs and symptoms suggestive of sepsis within 48 h of ICU admission were excluded to ensure that only incident cases of sepsis developing during ICU stay were evaluated. Patients with hematological malignancies, primary or acquired immunodeficiency disorders, and those receiving immunosuppressive therapy were excluded because these conditions can significantly influence leukocyte kinetics and inflammatory biomarkers. In addition, patients who developed sepsis but died within 24 h of diagnosis and patients who did not develop sepsis but expired within seven days of ICU admission were excluded due to insufficient follow-up data.

Baseline demographic and clinical data, including age, sex, body mass index, primary diagnosis, comorbidities, and Sequential Organ Failure Assessment (SOFA) scores, were extracted from hospital records [9]. Laboratory investigations performed within 24 hours of ICU admission were recorded for all participants. These investigations included complete blood count with IG#, IG%, total white blood cell count, neutrophil percentage, platelet count, hemoglobin concentration, serum creatinine, total bilirubin, serum lactate, PCT, CRP, and arterial blood gas parameters.

Immature granulocyte parameters were measured using an automated hematology analyzer, Sysmex XN-1000 (Sysmex Corporation, Kobe, Japan), which provides automated quantification of immature granulocytes as part of routine complete blood count [10]. Serum PCT levels were determined using a Cobas e411 immunoassay analyzer (Roche Diagnostics International Ltd., Rotkreuz, Switzerland) using electrochemiluminescence immunoassay technology [11]. Serum CRP concentrations were measured using an immunoturbidimetric assay according to the manufacturer's instructions and the standard laboratory protocols of the institutional central laboratory. Blood, urine, pus, and other body fluid culture reports were retrieved when clinically indicated. Microbiological investigations were performed according to the guidelines of the Indian Council of Medical Research for Good Clinical and Laboratory Practices [12]. Imaging findings and microbiological results were reviewed to identify the probable sources of infection.

All patients received standard treatment in accordance with institutional ICU protocols and were evaluated daily by the attending critical care team. Clinical and laboratory data were monitored for a maximum period of seven days following ICU admission or until the occurrence of sepsis, discharge, or death, whichever occurred earlier. Sepsis was defined according to the Sepsis-3 criteria as suspected or documented infection associated with acute organ dysfunction, represented by an increase in the SOFA score of at least two points from baseline. Patients who fulfilled the Sepsis-3 criteria during the observation period were classified into the sepsis group, whereas those who did not develop sepsis during the follow-up period were assigned to the no-sepsis group [13].

Sample size estimation

The sample size was estimated a priori using G*Power software version 3.1.9.7 (Heinrich Heine University Düsseldorf, Düsseldorf, Germany). Based on a previously reported sensitivity of approximately 81% for immature granulocyte parameters in sepsis diagnosis, a minimum sample size of 152 patients was calculated at a confidence level of 95% and statistical power of 80% [14]. To compensate for incomplete records and potential exclusion, the final target sample size was increased to 178 patients.

Blinding and data verification

To minimize information bias and ensure data accuracy, all eligible patient records were independently reviewed by two investigators affiliated with different academic institutions (PS, JS). Any discrepancies identified during data extraction were independently reviewed by a third investigator (AM), and the final decision was reached through consensus among the reviewers. After completion of data verification, the final anonymized dataset was provided to an independent biostatistician from a separate institution (AY) who was not involved in patient recruitment, clinical management, or data collection. Statistical analyses were performed independently in accordance with the predefined statistical analysis plan.

Data analysis

Statistical analysis was performed using the Statistical Package for the Social Sciences (SPSS) software (version 23.0, IBM Corp., Armonk, NY, USA). Continuous variables were assessed for normality and expressed as mean ± standard deviation or median with interquartile range as appropriate. Categorical variables are presented as frequencies and percentages. Comparisons between the sepsis and no-sepsis groups were performed using the independent samples Student’s t-test for normally distributed variables and Mann-Whitney U test for non-normally distributed variables. The Chi-square test was used to compare categorical variables. Receiver operating characteristic (ROC) curve analysis was performed to evaluate the diagnostic performance of IG#, IG%, PCT, and CRP levels. The area under the ROC curve (AUC), with corresponding 95% confidence intervals, was calculated for each biomarker. Optimal cut-off values were determined using Youden’s index, and the corresponding sensitivity, specificity, positive predictive value, negative predictive value, positive likelihood ratio, and negative likelihood ratio were calculated. Differences were considered statistically significant at p < 0.05.

## Results

A total of 178 critically ill patients admitted to the medical ICU were included in the study. Among them, 72 patients (40.4%) developed sepsis during the observation period and were categorized into the sepsis group, whereas 106 patients (59.6%) did not develop sepsis and constituted the no-sepsis group. The baseline characteristics of the study population are presented in Table 1. The mean age of patients in the sepsis group was 52.4 ± 14.6 years compared with 48.7 ± 15.2 years in the no-sepsis group, with no statistically significant difference (p = 0.087). Similarly, the sex distribution, body mass index, primary diagnosis, and comorbidities were comparable between the groups (p > 0.05). However, patients who developed sepsis had significantly higher baseline SOFA scores (5.8 ± 2.1 vs. 4.9 ± 1.9; p = 0.003) and a longer ICU stay (median 9 (IQR: 6-14) days vs. 7 (IQR: 5-11) days; p = 0.018).

**Table 1:** Baseline characteristics of the study participants Values are presented as mean ± standard deviation, median (interquartile range), or frequency (percentage), as appropriate. χ² = Chi-square test was used for categorical variables, t = Independent-samples t-test for normally distributed continuous variables, U = Mann–Whitney U test for non-normally distributed continuous variables. df = degree of freedom, *p-value < 0.05 was considered statistically significant. CKD: chronic kidney disease, COPD: chronic obstructive pulmonary disease, SOFA: Sequential Organ Failure Assessment, BMI: body mass index, IQR: interquartile range.

Variable		Sepsis group (n = 72)	No-sepsis group (n = 106)	Total (N = 178)	Test value (df)	p-value
Age, mean ± SD	Years	52.4 ± 14.6	48.7 ± 15.2	50.2 ± 15.0	t = 1.31 (176)	0.087
Sex, n (%)	Male	46 (63.9)	62 (58.5)	108 (60.7)	*χ²* = 0.87 (1)	0.472
	Female	26 (36.1)	44 (41.5)	70 (39.3)		
BMI, mean ± SD	kg/m²),	23.8 ± 3.9	24.2 ± 4.1	24.0 ± 4.0	t = 1.08 (176)	0.524
Primary diagnosis, n (%)	Respiratory failure	24 (33.3)	32 (30.2)	56 (31.5)	*χ² = *0.91 (4)	0.641
	Acute kidney injury	18 (25.0)	28 (26.4)	46 (25.8)		
	Hepatic failure	10 (13.9)	14 (13.2)	24 (13.5)		
	Cardiac (non-septic)	11 (15.3)	19 (17.9)	30 (16.9)		
	Other	9 (12.5)	13 (12.3)	22 (12.4)		
Comorbidities, n (%)	Diabetes mellitus	29 (40.3)	38 (35.8)	67 (37.6)	*χ² = *1.16 (3)	0.559
	Hypertension	31 (43.1)	42 (39.6)	73 (41.0)		
	CKD (non-dialysis)	14 (19.4)	18 (17.0)	32 (18.0)		
	COPD	12 (16.7)	15 (14.2)	27 (15.2)		
ICU admission, mean ± SD	SOFA	5.8 ± 2.1	4.9 ± 1.9	5.3 ± 2.0	t = 4.25 (176)	0.003*
Length of ICU stay, median (IQR)	days	9 (6–14)	7 (5–11)	8 (5–12)	U = 2412	0.018*

A comparison of laboratory biomarkers between the two groups is summarized in Table 2. The median IG# and IG% were significantly higher among patients who developed sepsis than among those who did not. The median IG# was 0.09 × 10⁹/L (IQR: 0.05-0.16) in the sepsis group compared with 0.02 × 10⁹/L (IQR: 0.01-0.04) in the no-sepsis group (p < 0.001). Similarly, the median IG% was 1.72% (IQR: 1.10-2.55) in sepsis patients and 0.48% (IQR: 0.22-0.82) in non-sepsis patients (p < 0.001). Procalcitonin levels were also markedly elevated in the sepsis group (4.18 (1.82-11.60) ng/mL vs. 0.35 (0.14-0.82) ng/mL; p < 0.001). Significant differences were also observed in lactate, CRP, serum creatinine, total bilirubin, white blood cell count, neutrophil percentage, platelet count, and SOFA score (all p < 0.05). Hemoglobin levels did not differ significantly between the groups (p = 0.060).

**Table 2:** Comparison of sepsis biomarkers between groups. Values are presented as mean ± standard deviation or median (interquartile range), as appropriate. U: Mann–Whitney U test was used for non-normally distributed variables, t: Independent-samples t-test was used for normally distributed variables, Effect size (r) was calculated from the standardized test statistic, *p < 0.05 was considered statistically significant. IG#: immature granulocyte count, IG%: immature granulocyte percentage, PCT: procalcitonin, CRP: C-reactive protein, WBC: white blood cell, SOFA: Sequential Organ Failure Assessment.

Biomarker	Measure	Sepsis (n = 72)	No-sepsis (n = 106)	Test statistic	p-value	Effect size (r)
IG# (×10⁹/L)	Median (IQR)	0.09 (0.05–0.16)	0.02 (0.01–0.04)	U=1624	< 0.001*	0.63
IG% (%)	Median (IQR)	1.72 (1.10–2.55)	0.48 (0.22–0.82)	U=1587	< 0.001*	0.62
PCT (ng/mL)	Median (IQR)	4.18 (1.82–11.60)	0.35 (0.14–0.82)	U=1702	< 0.001*	0.65
Lactate (mmol/L)	Median (IQR)	3.2 (2.1–5.4)	1.8 (1.2–2.8)	U=2108	< 0.001*	0.46
CRP (mg/L)	Median (IQR)	108 (78–148)	58 (32–90)	U=2682	< 0.001*	0.35
Serum creatinine (mg/dL)	Median (IQR)	2.4 (1.4–4.2)	1.3 (0.9–2.0)	U=2456	< 0.001*	0.40
Total bilirubin (mg/dL)	Median (IQR)	2.1 (1.0–4.4)	1.1 (0.6–2.0)	U=2841	< 0.001*	0.31
WBC count (×10⁹/L)	Median (IQR)	14.8 (10.2–20.4)	9.6 (7.2–13.1)	U=2234	< 0.001*	0.44
Neutrophil (%)	Mean ± SD	82.4 ± 7.2	72.8 ± 8.6	t=7.24	< 0.001	0.43
Hemoglobin (g/dL)	Median (IQR)	9.8 (8.4–11.2)	10.4 (9.0–11.8)	U=4608	0.060*	0.14
Platelet count (×10³/µL)	Median (IQR)	162 (104–226)	198 (148–264)	U=5082	0.005*	0.21
SOFA score (day of sepsis/day 7)	Mean ± SD	8.2 ± 2.4	5.3 ± 1.8	t=8.06	< 0.001*	0.52

The diagnostic performances of the evaluated biomarkers are presented in Table 3. An IG% cut-off value of ≥0.80% demonstrated a sensitivity of 86.1% and specificity of 78.3%, with a positive likelihood ratio of 3.96 and a negative likelihood ratio of 0.18. IG# at a threshold of ≥0.055 × 10⁹/L yielded a sensitivity of 83.3% and a specificity of 76.4%. PCT at a cut-off value of ≥0.90 ng/mL showed a sensitivity of 80.6% and a specificity of 74.5%, whereas CRP exhibited comparatively lower sensitivity (69.4%) and specificity (62.3%). The combined use of IG% and PCT provided the highest diagnostic accuracy, achieving a sensitivity of 90.3%, specificity of 82.1%, positive predictive value of 77.4%, negative predictive value of 92.9%, and the highest Youden’s index (0.724).

**Table 3:** Diagnostic accuracy of biomarkers for sepsis detection. Cut-off values were determined using Youden’s index. Diagnostic accuracy measures are presented as sensitivity, specificity, positive predictive value, negative predictive value, positive likelihood ratio, negative likelihood ratio, and Youden’s index. PPV: positive predictive value, NPV: negative predictive value, LR: likelihood ratio, IG#:  immature granulocyte count, IG%: immature granulocyte percentage, PCT: procalcitonin, CRP: C-reactive protein.

Biomarker (Cut-off)	Sensitivity (%)	Specificity (%)	PPV (%)	NPV (%)	+ LR	− LR	Youden's index
IG% ≥0.80%	86.1	78.3	72.1	89.8	3.96	0.18	0.644
IG# ≥0.055×10⁹/L	83.3	76.4	70.0	87.9	3.53	0.22	0.597
PCT ≥0.90 ng/mL	80.6	74.5	66.9	85.7	3.16	0.26	0.551
CRP ≥80 mg/L	69.4	62.3	55.1	75.0	1.84	0.49	0.317
IG% + PCT (combined)	90.3	82.1	77.4	92.9	5.04	0.12	0.724

**Figure 1:**
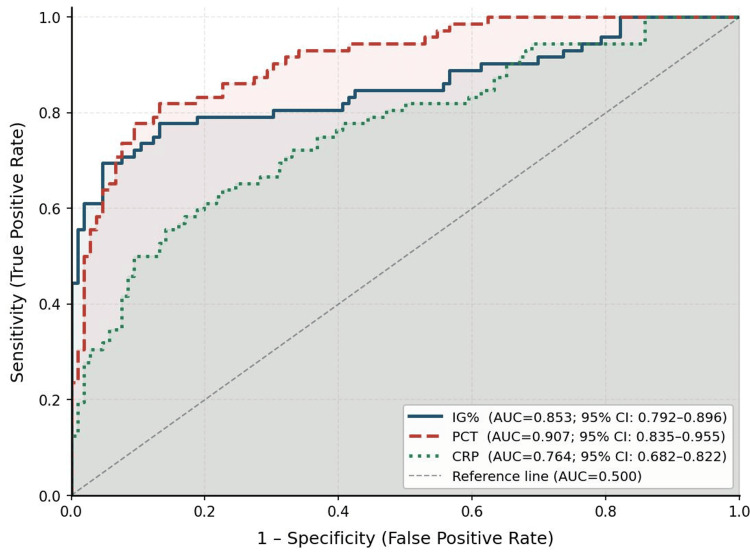
Receiver operating characteristic curves of immature granulocyte percentage, procalcitonin, and C-reactive protein for sepsis prediction. Receiver operating characteristic curves illustrating the diagnostic performance of immature granulocyte percentage, procalcitonin, and C-reactive protein for early sepsis detection among 178 intensive care unit patients. The diagonal reference line represents the performance of a non-discriminatory test. Areas under the curve are presented with corresponding 95% confidence intervals. ROC: receiver operating characteristic, AUC: areas under the ROC curves, CI: confidence interval, IG%: immature granulocyte percentage, PCT: procalcitonin, CRP: C-reactive protein.

The ROC curve analysis results are shown in Table 4 and Figure 1. Among the individual biomarkers, IG% demonstrated the highest diagnostic accuracy, with an area under the curve (AUC) of 0.853 (95% CI: 0.792-0.896), indicating good discriminative ability for early sepsis detection. IG# also showed good performance with an AUC of 0.841 (95% CI: 0.779-0.888). PCT yielded an AUC of 0.822 (95% CI: 0.757-0.875), whereas CRP demonstrated fair diagnostic performance with an AUC of 0.764 (95% CI: 0.692-0.826). All ROC analyses were statistically significant (p < 0.001).

**Table 4:** Receiver operating characteristic (ROC) curve analysis of biomarkers for sepsis prediction. ROC analysis was performed to assess the diagnostic performance of biomarkers. The area under the curve is presented with 95% confidence intervals. Optimal cut-off values were determined using Youden’s index. A *p-value < 0.05 was considered statistically significant. AUC: area under the curve; CI: confidence interval, ROC: receiver operating characteristic, IG#: immature granulocyte count, IG%: immature granulocyte percentage, PCT: procalcitonin, CRP: C-reactive protein.

Biomarker	AUC	95% CI	Optimal cut-off	Sensitivity (%)	Specificity (%)	p-value
IG%	0.853	0.792–0.896	0.80%	86.1	78.3	< 0.001*
IG#	0.841	0.779–0.888	0.055×10⁹/L	83.3	76.4	< 0.001*
PCT	0.822	0.757–0.875	0.90 ng/mL	80.6	74.5	< 0.001*
CRP	0.764	0.692–0.826	80 mg/L	69.4	62.3	< 0.001*

## Discussion

The present study evaluated the diagnostic utility of immature granulocyte parameters (IG% and IG#) for the early detection of sepsis in critically ill patients and compared their performance with that of established biomarkers, including PCT and CRP. The findings demonstrated that patients who developed sepsis exhibited significantly higher levels of IG%, IG#, PCT, CRP, lactate, serum creatinine, total bilirubin, white blood cell count, neutrophil percentage, and SOFA scores than those who did not develop sepsis. These observations support the concept that sepsis is characterized by an exaggerated inflammatory response associated with increased bone marrow activation and the accelerated release of immature granulocytes into the peripheral circulation.

Among the evaluated biomarkers, IG% emerged as one of the most promising indicators of early sepsis. The median IG% was significantly higher in the sepsis group than in the no-sepsis group, with a large effect size, suggesting a strong association between elevated immature granulocyte levels and sepsis development. Similar findings were reported by Bhansaly et al., who observed significantly increased IGs# among patients with sepsis and concluded that IG measurements may serve as early markers of infection before the appearance of overt clinical manifestations [14]. Similarly, Ayres et al. demonstrated that elevated IGs# are associated with systemic inflammatory states and could assist clinicians in the early identification of critically ill patients at risk of sepsis [15]. Patients younger than 15 years were excluded in our study because pediatric sepsis differs from adolescent and adult sepsis in terms of immune response, hematological reference ranges, and immature granulocyte kinetics, which could influence biomarker interpretation and reduce the homogeneity of the study population.

Cimenti et al. reported significantly higher IGs# and immature myeloid information in neonates with early-onset sepsis than those in healthy controls. Their findings suggest that automated immature granulocyte assessment may facilitate early recognition of sepsis, which is in agreement with the present study demonstrating the diagnostic utility of IG parameters for sepsis detection in critically ill patients [16].

The ROC analysis further supported the diagnostic usefulness of immature granulocytes. In the present study, IG% demonstrated good discriminatory ability, with an AUC of 0.853, whereas IG# showed a comparable AUC of 0.841. Nierhaus et al. reported that IGs# showed a strong predictive value for infection and sepsis in critically ill patients and were superior to several conventional hematological parameters [17]. The ability of automated hematology analyzers to rapidly provide IG values at no additional cost further enhances their clinical applicability in emergency and ICU settings. Porizka et al. observed that the diagnostic ability of IG% was similar to that of PCT in identifying sepsis in postoperative cardiac surgery patients [18]. Furthermore, combining IG% with PCT improved the overall diagnostic performance, which is consistent with the present study, where the combined use of IG% and PCT yielded the highest sensitivity, specificity, and AUC.

PCT also demonstrated an excellent diagnostic performance in this study. Septic patients had markedly elevated PCT concentrations compared to patients without sepsis, and ROC analysis yielded an AUC of 0.822. These results are in agreement with the findings of Wacker et al., who identified PCT as one of the most reliable biomarkers of bacterial sepsis [19]. Elevated PCT concentrations reflect systemic bacterial infection and are less influenced by non-infectious inflammatory conditions than CRP levels. Consequently, PCT is an important biomarker for the diagnosis and therapeutic monitoring of sepsis. Nevertheless, PCT testing is associated with higher laboratory costs and may not be readily available in all healthcare settings, particularly in resource-limited settings.

Compared with the IG parameters and PCT, CRP exhibited a relatively lower diagnostic performance, with an AUC of 0.764 and lower sensitivity and specificity. Although CRP remains one of the most commonly used inflammatory biomarkers, its lack of specificity limits its usefulness in distinguishing infectious from non-infectious causes of systemic inflammation. Similar observations have been reported by Lobo et al., who noted that CRP levels increase in a variety of inflammatory conditions and, therefore, should not be used as an isolated marker for sepsis diagnosis [20]. The present findings reinforce the view that CRP level may be more useful when interpreted alongside other laboratory and clinical parameters.

Gucyetmez and Atalan found that although several routine hematological parameters had limited discriminative ability, combining CRP with lymphocyte and platelet counts substantially improved sepsis prediction [21]. These findings support the results of the present study, in which the combination of biomarkers, particularly IG% and PCT, provided superior diagnostic accuracy compared to individual markers. Lalmuanpuia et al. reported significantly elevated inflammatory biomarkers, including CRP, PCT, white blood cell count, and neutrophil-to-lymphocyte ratio, in patients with odontogenic infections, highlighting the clinical value of hematological and inflammatory markers in the early identification and assessment of infection severity [22].

One of the most important findings of this study was the improved diagnostic performance achieved by combining the IG% and PCT. The combined model yielded the highest sensitivity, specificity, positive predictive value, negative predictive value, and ROC area (AUC = 0.914), indicating excellent discrimination between patients with and without sepsis. This suggests that immature granulocyte assessment and PCT levels provide complementary information regarding host inflammatory and infectious responses. Similar benefits of combining biomarkers have been described by Pierrakos and Vincent, who emphasized that no single biomarker possesses sufficient accuracy for all clinical situations and that multimarker approaches generally improve diagnostic confidence [23].

The significantly higher SOFA scores and longer ICU stay observed among patients with sepsis further validated the clinical relevance of the study findings. These results are consistent with the Sepsis-3 framework, which recognizes organ dysfunction as a defining feature of sepsis and identifies the SOFA score as a valuable prognostic and diagnostic tool [9]. Collectively, the present findings indicate that immature granulocyte parameters, particularly IG%, are valuable biomarkers for early sepsis detection and may offer diagnostic performance comparable to that of procalcitonin. Their rapid availability through routine complete blood count analysis makes them particularly attractive in critical care settings. Furthermore, the combination of IG% and PCT appears to provide the greatest diagnostic accuracy and may facilitate early recognition and intervention in patients at risk of sepsis.

This study had certain limitations that should be considered when interpreting the findings. First, its retrospective observational design may be subject to selection bias, which limits the ability to establish causal relationships. Second, the study was conducted at a single tertiary care center, which may restrict the generalizability of the results to other healthcare settings and patient populations. Third, biomarker measurements were based on routinely available clinical data, and serial trends in immature granulocyte parameters beyond the observation period were not evaluated. Additionally, potential confounding factors related to underlying illnesses, variations in infection sources, and treatment interventions could have influenced biomarker levels. Another limitation is that the clinical applicability of immature granulocyte parameters depends on the availability of automated hematology analyzers capable of reporting IG values. In laboratories lacking such instrumentation, immature granulocytes must be identified by manual differential leukocyte counting, which is more time-consuming, resource-intensive, and subject to observer variability, potentially limiting the widespread implementation of this biomarker. Finally, although the sample size was adequate for analysis, larger multicenter prospective studies are required to validate the diagnostic utility and optimal cut-off values of immature granulocyte parameters in diverse critically ill populations.

## Conclusions

The present study demonstrated that immature granulocyte parameters, particularly IG%, are reliable and effective biomarkers for early detection of sepsis in critically ill patients. IG% showed good diagnostic accuracy, comparable to PCT, and superior performance to CRP. Furthermore, the combination of IG% and PCT showed the highest sensitivity, specificity, and overall diagnostic accuracy. As immature granulocyte measurements are rapidly available through routine hematological analysis, they represent a practical and cost-effective adjuncts for early sepsis diagnosis and timely clinical intervention.

## References

[1] Jarczak D, Kluge S, Nierhaus A (2021). Sepsis-pathophysiology and therapeutic concepts. Front Med (Lausanne).

[2] Caraballo C, Jaimes F (2019). Organ dysfunction in sepsis: an ominous trajectory from infection to death. Yale J Biol Med.

[3] Daud M, Khan MB, Qudrat QU (2024). Role of C-reactive protein and procalcitonin in early diagnostic accuracy and their prognostic significance in sepsis. Cureus.

[4] Tan M, Lu Y, Jiang H, Zhang L (2019). The diagnostic accuracy of procalcitonin and C-reactive protein for sepsis: a systematic review and meta-analysis. J Cell Biochem.

[5] Prabu NR, Patil VP (2020). Is immature granulocyte count a potential prognostic marker for upper gastrointestinal tract bleeding? A new road to explore. Indian J Crit Care Med.

[6] V L, K K, E Y, M S (2024). The clinical utility of automated immature granulocyte measurement in the early diagnosis of bacteremia. Cureus.

[7] Senthilnayagam B, Kumar T, Sukumaran J, M J, Rao K R (2012). Automated measurement of immature granulocytes: performance characteristics and utility in routine clinical practice. Patholog Res Int.

[8] Nigro KG, O'Riordan M, Molloy EJ, Walsh MC, Sandhaus LM (2005). Performance of an automated immature granulocyte count as a predictor of neonatal sepsis. Am J Clin Pathol.

[9] Moreno R, Rhodes A, Piquilloud L (2023). The Sequential Organ Failure Assessment (SOFA) Score: has the time come for an update?. Crit Care.

[10] Ansari-Lari MA, Kickler TS, Borowitz MJ (2003). Immature granulocyte measurement using the Sysmex XE-2100. Relationship to infection and sepsis. Am J Clin Pathol.

[11] Huang J, Zu Y, Zhang L, Cui W (2024). Progress in procalcitonin detection based on immunoassay. Research (Wash D C).

[12] Pahuja M (2021). Good clinical laboratory practices guidelines (2nd edition). https://ethics.ncdirindia.org/asset/pdf/GCLP_Guidelines_2020_Final.pdf.

[13] Singer M, Deutschman CS, Seymour CW (2016). The Third International Consensus Definitions for Sepsis and Septic Shock (Sepsis-3). JAMA.

[14] Bhansaly P, Mehta S, Sharma N, Gupta E, Mehta S, Gupta S (2022). Evaluation of immature granulocyte count as the earliest biomarker for sepsis. Indian J Crit Care Med.

[15] Ayres LS, Sgnaolin V, Munhoz TP (2019). Immature granulocytes index as early marker of sepsis. Int J Lab Hematol.

[16] Cimenti C, Erwa W, Herkner KR, Kasper DC, Müller W, Resch B (2012). The predictive value of immature granulocyte count and immature myeloid information in the diagnosis of neonatal sepsis. Clin Chem Lab Med.

[17] Nierhaus A, Klatte S, Linssen J (2013). Revisiting the white blood cell count: immature granulocytes count as a diagnostic marker to discriminate between SIRS and sepsis-a prospective, observational study. BMC Immunol.

[18] Porizka M, Volny L, Kopecky P, Kunstyr J, Waldauf P, Balik M (2019). Immature granulocytes as a sepsis predictor in patients undergoing cardiac surgery. Interact Cardiovasc Thorac Surg.

[19] Wacker C, Prkno A, Brunkhorst FM, Schlattmann P (2013). Procalcitonin as a diagnostic marker for sepsis: a systematic review and meta-analysis. Lancet Infect Dis.

[20] Lobo SM, Lobo FR, Bota DP, Lopes-Ferreira F, Soliman HM, Mélot C, Vincent JL (2003). C-reactive protein levels correlate with mortality and organ failure in critically ill patients. Chest.

[21] Gucyetmez B, Atalan HK (2016). C-reactive protein and hemogram parameters for the non-sepsis systemic inflammatory response syndrome and sepsis: what do they mean?. PLoS One.

[22] Lalmuanpuia C, Kaushik K, Kathuria B (2026). Inflammatory biomarker profiles in odontogenic infections: evaluation of C-reactive protein (CRP), procalcitonin, WBC count, and neutrophil-to-lymphocyte ratio. Cureus.

[23] Pierrakos C, Vincent JL (2010). Sepsis biomarkers: a review. Crit Care.

